# From hashtags to ballots: Conceptualizing political influencers and evaluating their impact on election outcomes

**DOI:** 10.1371/journal.pone.0321592

**Published:** 2025-05-07

**Authors:** Carsten Schwemmer, Magdalena Riedl

**Affiliations:** 1 Professorship of Computational Social Sciences, Department of Sociology, LMU Munich, Bavaria, Germany; 2 Chair for General and Theoretical Sociology, Institute of Sociology, University of Jena, Jena, Thuringia, Germany; University of Zurich Irchel Campus: Universitat Zurich Standort Irchel, SWITZERLAND

## Abstract

Social media influencers have evolved beyond lifestyle content, with a new subset - political influencers - rising to prominence in shaping political discourse. This paper seeks to highlight important streams of literature for defining political influencers and examining their impact on elections. We provide guidelines for conceptualizing political influencers, offering key operational definitions and decisions for future studies. Using the 2021 German federal elections as a case study, we analyze the behavior of political influencers on Instagram, focusing on the blend of political and commercial content. Our analysis reveals that political influencers shift their focus from product promotion to political messaging and support or disapproval of political entities in the lead-up to the elections. Based on post-survey election data, we further assess the relevance of influencers for voting decisions, identifying individual characteristics, such as age, associated with following influencers. We find that approximately six percent of respondents found influencer-produced content helpful in determining their voting decisions. These insights shed light on the growing importance of political influencers in electoral dynamics, providing a foundation for further exploration of their long-term political impact.

## 1. Introduction

With most of the global population accessing smartphones and the internet, social media has revolutionized how we communicate and interact with others. On average, people spend more than two hours daily on social media [1,2]. Among entertainment as one of the main reasons for social media use, news consumption and political engagement are becoming increasingly important [3,4]. Access to social media not only connects people and increases real-time information flow but also bears the potential for virtually anybody with access to a smartphone to reach huge audiences. Social media Influencers are experts at maximizing content dissemination on online platforms. At the same time, they can affect social, political, and economic attitudes and behavior, such as purchasing decisions, due to their reliance on product promotion for monetization [5,6]. Influencers are also regarded as opinion leaders who cooperate with brands to market products related to fashion, fitness, food, and gaming via electronic word-of-mouth mechanisms.

In recent years, a subgroup of influencers has grown in size and relevance: political influencers [7]. These individuals increasingly include political content in their regular narrative to disseminate content related to political topics, such as climate change, and political events, such as elections [3]. In turn, they have become an increasingly important aspect of political discourse and also have started to play a significant role in election campaigns. The election campaign of presidential candidate Mike Bloomberg, for instance, offered $150 for influencers to spread election advertising via social media [8], while at the same time, German parties were criticized for processing personal data from the Facebook platform and creating extensive voter profiles without explicit and informed consent.

Political influencers are now recognized as a new class of opinion leaders who not only shape consumer preferences but also have the potential to sway public opinions and political decisions [9–11]. Their influence can be positive, such as advocating for political participation, and negative, for instance, when promoting misinformation. As Harff et al. [12] suggest, political content shared by influencers often garners significant attention, highlighting their capacity to engage audiences with meaningful topics. At the same time, influencers must carefully balance their political messaging with their commercial interests, as studies indicate that maintaining audience trust and avoiding controversial topics are crucial for sustaining collaborations [4].

Moreover, their commercial activities, such as product placements, raise important questions regarding authenticity and trustworthiness, particularly when intertwined with political content. This dual role—amplifying political messages while operating within a marketplace framework—underscores the growing intersection of marketing and politics, as outlined by Gonzalez et al. [13].

Despite a growing number of initial studies beginning to examine political influencers in particular [7,14,15], the literature still covers only partial insights in their actual impact on political discourses and election outcomes. In particular, little is known about both the prevalence of political content and advertisements in influencers posts during electoral campaigns. It is further unclear to what extent content of influencers is relevant for voters’ decision-making in such periods. In addition, the term ‘political influencers’ does not relate to a clear-cut group of individuals, so scholars use a wide range of operationalizations and concept definitions. Our paper seeks to address these conceptual and empirical research gaps. First, we outline a theoretical background for conceptualizing and a guideline for operationalizing political influencers. In turn, we outline several key decisions that scholars wishing to study this phenomenon must make. Second, we demonstrate the application of these guidelines in a case study on political influencers and the German federal elections in 2021. Using Instagram data, we show that - similar to conventional influencers - political influencers seemingly integrate product promotions with substantial political content in their posts. We also find that they resort to less advertising, more political content, and more support and disapproval of political entities shortly before the election. Finally, we use self-administered survey data to examine the helpfulness of influencers’ content for electoral decision-making of German citizens. Our results suggest that while the impact of influencers is relatively weak compared to other factors, such as conversations with friends or relatives, it still translates into a substantial number of citizens for whom influencers’ content is considered when making voting decisions.

## 2. Conceptualizing political influencers

While the phenomenon of political influencers is quite recent, scholars interested in studying political influencers can refer to two areas for which past research already covers a lot of ground: 1) research on political participation and communication on social media and 2) research on conventional (non-political) influencers. Reviewing the predominant theories and empirical findings from both streams of literature is beyond the scope of this paper. Instead, we briefly outline and bridge insights from both areas to provide a starting point for future scholars to expand upon.

### 2.1. Political participation and communication on social media

Social media has fundamentally changed the way people participate in political discourse. By providing an online platform for individuals to engage in political discussions and express their political views, social media enables political communication globally, at scale, and in rapid succession. Social media provides new opportunities not only for ordinary citizens but also for journalists and politicians. Journalistic YouTubers, for example, aim to push journalism toward younger target groups, establish entertaining presentation styles, and strongly focus on audience interactions [16]. Politicians use social media for election campaigning [17] and to share their messages across different target groups [18]. However, in the remainder of this paper, we focus on political communication from the perspective of citizens and potential voters, as this also reflects the audience of influencers. Among this audience, political participation comes in various forms and shapes, including, for instance, liking and sharing political content and engaging in political discussions. Socio-demographics and other factors, in turn, affect the kind of people who are more likely to engage in political discourses on social media. For example, young people, in particular, are more likely to use social media as it is also their primary source of news and information [19].

On the one hand, these possibilities for online political communication go hand in hand with the promise of lower-threshold opportunities and the associated increase in political equality. Some studies have shown that political participation on social media can have a significant impact on political outcomes, particularly in terms of voter turnout and political efficacy [20,21]. However, access to social media by itself does not necessarily lead to an increase in political involvement, as a large part of the increased participation is due to the self-selection processes of politically active citizens. As other studies suggest, it is primarily people who have already been active offline who are participating in the political discourse online [22,23]. A key question, therefore, is how to engage citizens who are active on social media but not for political reasons. According to a study in Germany, reaching younger voters requires authentic and personal content, explaining politics in simple terms, and adjusting to communication cultures and specifics of online platforms [24]. Social media influencers excel at these requirements, bridging the connection between online political communication and authentic opinion leaders.

### 2.2. Influencers as authentic opinion leaders

While there are many definitions of influencers, especially in the marketing literature, a dominant definition across scientific fields has yet to be established. Abidin, for example, defines influencers as

“everyday, ordinary Internet users who accumulate a relatively large following on blogs and social media through the textual and visual narration of their personal lives and lifestyles, engage with their following in digital and physical spaces, and monetize their following by integrating ‘advertorials’ into their blog or social media posts. [...] It is a functional attribution that organizations apply to social media users such as bloggers, YouTubers, Instagrammers, etc., that are ascribed the ability to influence the organization’s stakeholders and thus become relevant to the organization.” [25]

On the one hand, it is clear from this definition that influencers present themselves as “ordinary people,” thus, a distinction is made between them and celebrities, such as film stars or well-known athletes [26]. On the other hand, influencers are characterized by the fact that they incorporate product placements or advertisements into their everyday narrative and thus monetize their profile. As ordinary people, influencers report on their everyday lives and attract viewers to their profiles through the (supposedly) authentic portrayal of their lives. It is precisely this authenticity that influencers embody that is the core of their recipe for success [27,28]. The impression of authenticity conveyed is presented in research as a decisive success factor [29]. However, many so-called “micro-influencers” with a comparatively small number of followers do not operate their profile full-time and see their income from possible collaborations as a sideline or hobby.

Influencers are frequently characterized by their authenticity and interactivity, leading to ongoing debates about their role as authentic opinion leaders. [3,27,28]. In our work, we follow the established concept of Paul Lazarsfeld and consider opinion leaders as individuals who are particularly influential in shaping the opinions and behaviors of others within their social networks. Certain knowledge and expertise are attributed to them, which enables opinion leaders to communicate their opinions and ideas to others effectively [30]. In the 1940s, when the concept gained popularity in the academic debate, Lazarsfeld and others suggested that these individuals play an important role in shaping public opinion and behavior through two-step-communication flow. The two-step flow of communication theory posits that media messages influence the public indirectly, primarily through opinion leaders who first interpret and then relay these messages to others [31]. Although the concept of opinion leadership is not new, through social media and the associated communication options, it gains new relevance. While the original concept refers to face-to-face interactions, i.e., conversations with friends and acquaintances, digital opinion leaders differ in that they interact with their followers online. Unlike traditional opinion leaders, influencers can reach a much larger audience due to algorithm-driven visibility and network effects, yet they still cultivate perceptions of authenticity and peer-like relationships through parasocial interactions [27,32]. Potential opinion leaders can now build a greater network and reach a higher audience (respectively, higher influence). Recent research also suggests that the dissemination of information through social media is evolving into a multi-step flow with different kinds of opinion leaders, adopters, and influencers [33].

However, opinion leadership in isolation is insufficient for connecting political communication and influencers to develop a unified definition of political influencers. Within the limited but growing share of studies on political influencers, quite different concept definitions can be found [11,13]. To give one example from the introduction of a special issue on the topic: “We define political influencers as content creators that endorse a political position, social cause, or candidate through media that they produce and/or share on a given social media platform” [7]. The authors further outline that this definition can also include politicians and journalists. Similarly, Bause speaks of political social media influencers. She also refers to the concept of opinion leadership in her definition and describes influencers as “as self-created personal brands” [27]*.* Depending on the focus of a study, this rather broad definition of political influencers may be an excellent starting point. For other studies, particularly those with a strong connection to the concept of influencers as ordinary citizens establishing themselves as authentic opinion leaders, a more restrictive conceptualization of political influencers may be necessary [33]. Such conceptualizations may, for instance, exclude established elites such as party politicians or popular journalists as entities that should be included in an appropriate sampling frame for empirically analyzing the content of political influencers. For our case study, we refer to Abidin’s definition and focus on ordinary users rather than journalists and politicians who share political content. This narrower conceptualization allows us to specifically analyze influencers as a distinct phenomenon characterized by their perceived authenticity, interactivity, and grassroots-level impact.

## 3. Operationalization and measurement of political influencers

**Fig 1:**
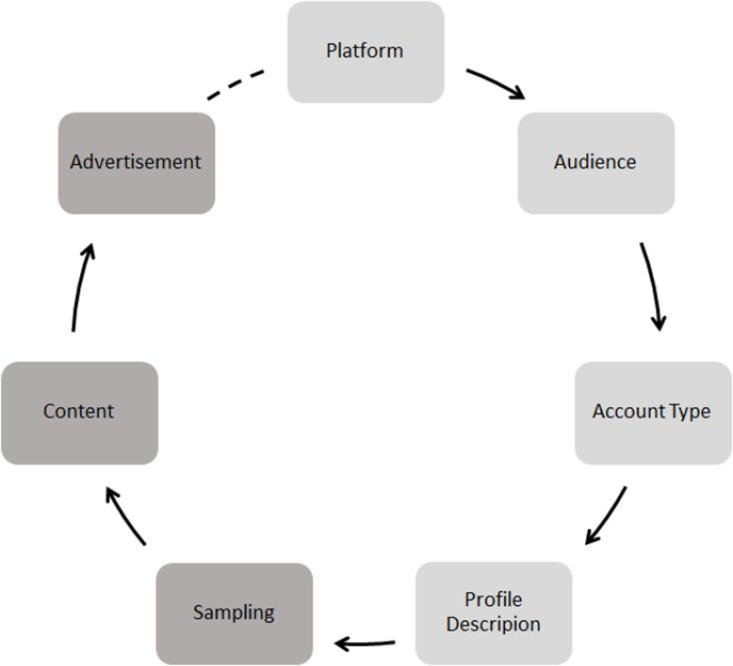
Circle flowchart for operationalizing a sample of political influencers and their generated content. Factors relevant before data collection are depicted in light gray, whereas post-data-collection factors are depicted in dark gray.

Following the conceptualization of what entities should be considered political influencers, scholars need an appropriate operationalization strategy for collecting and analyzing influencer content. In Fig 1, we propose a circle flowchart for this process and related options that must be considered. Throughout the remainder of this section, we will discuss the components of this flowchart and highlight several key decisions that scholars need to make along the way.

### Platform

One of the first decisions when studying influencers is what online platforms should be selected for data collection. This decision has significant implications, as social media platforms are tailored toward different target audiences, with varying user interfaces and varying models for maximizing engagement to accompany the underlying business models, which are predominantly based on advertisement revenue. At the same time, these affordances lead to some platforms being more popular among influencers than others. Besides decisions based on substantive criteria, accessing platform data is an additional technical hurdle challenging scholars who wish to study social media. While in principle, data from all platforms should be accessible to researchers via endeavors such as the Digital Service Act [34], in practice, data access is becoming more difficult. The situation about which application programming interfaces (APIs) for retrieving platform data are available for scientific use and at what cost is rapidly evolving. In the Post-API Age [35], most social media platforms do not provide means to access their content programmatically. This may lead to scholars applying data scraping procedures and violating terms of service, posing additional legal and ethical challenges [36].

### Audience

After identifying the platforms of interest, scholars must decide upon the audience political influencers need to have accumulated to be considered for further analysis. A simple categorization approach is based on the number of followers or subscribers, as it offers insights into the account’s reach. It indicates how many people are regularly exposed to its content. Profiles are often categorized into different tiers based on follower counts. It is important to note that while the number of followers has important consequences regarding the potential reach, influencers with smaller audiences can still achieve the status of microcelebrities [14,37] and use corresponding techniques to increase their credibility. Authenticity and credibility can be at least or even more important for political influencers than many followers [38]. Depending on the research question, it may also be relevant to consider the engagement rate as a selection criterion beyond the follower count. Engagement rate is a metric that measures the level of interaction and engagement users have with social media content. It considers actions like likes, comments, shares, and clicks, providing a more comprehensive view of user involvement beyond just likes [39]. This metric is valuable because it reflects engagement quality, audience sentiment, potential virality, and content effectiveness. By analyzing engagement rates, researchers can gain insights into user behavior and assess the impact of their social media strategies.

### Account type

While metrics such as the number of followers provide initial insights into the reach and sphere of visibility of influencers, many research questions require a deeper examination of their profiles. Therefore, the next step discussed focuses on the account type. While content creators prioritize self-produced content, which real individuals typically portray, other accounts are predominantly used to disseminate memes [40]. Another possibility is using accounts by multiple individuals, such as a couple of accounts or those representing organizations or events. Additionally, the rise of virtual characters, also known as AI influencers, is gaining momentum. AI influencers are virtual personas that engage with audiences on social media platforms, often mimicking human-like behavior and characteristics [41]. For research questions that primarily revolve around the content of a post or account, this distinction may be of secondary importance. However, when the focus is set on influencers as authentic human opinion leaders, this differentiation becomes significant and should be considered when selecting cases.

### Profile description

As mentioned above, there are many definitions of influencers that are shared across different areas. While some subsume politicians, journalists, or celebrities under the term influencer, some intentionally focus on ordinary internet users, bloggers, and content creators. Fischer et al., for example, follow the second line by only analyzing influencers without an “institutional background in the established media and/or political system” [42]. De Gregorio includes politicians and content creators but differentiates between three types: Politicians who present themselves as Social Media Influencers, Social Media Influencers who appear as political opinion leaders and Social Media Influencers turned politicians [43]. Depending on the conceptualization of political influencers, scholars may use external resources to identify interest profiles. For example, official social media accounts of public figures are often available in online repositories or curated datasets of past research. In any case, qualitative examinations of social media profile descriptions in conjunction with available content help identify political influencers and exclude entities not part of the desired research sample.

### Sampling

Once a sufficient set of influencer accounts is identified, scholars must decide upon an appropriate sampling strategy for capturing the content of interest. One of the biggest challenges when studying political influencers is that the target population of interest is in constant flux, making it hard to analyze a stable sample over time. Therefore, a number of related decisions have to be considered. For one, an important question is what kind of content should be sampled over what time period. Some content types, such as Instagram stories (see next paragraph), are usually only available for a limited time. Moreover, some content, such as hateful or xenophobic posts by right-wing influencers, may frequently be deleted from online platforms. Both cases require scholars to adjust their sampling frequency accordingly. Samples might also be adjusted in case of prolonged observation periods as some accounts of political influencers vanish, while new accounts matching the selection criteria may appear.

### Content

The content that is distributed on social media platforms varies greatly depending on the platform and offers users the opportunity to express themselves via text, images, and videos. The platforms are subject to constant change regarding which formats currently work best for reaching large audiences. One example is Instagram, which initially focused on images, leading to influencers mainly producing content favored by the platform’s algorithm [44]. Studies also show that images with faces achieve a higher reach than other image types [45]. In the meantime, however, Instagram has evolved and is increasingly promoting so-called reels, short videos that are given preferential treatment in the algorithm. The situation is similar on TikTok, with a strong focus on videos. For researchers working with social media content, this poses the challenge of working with different modalities (image, video, text, sound) and the constant need to follow trends about how content is produced and distributed by influencers. Content also includes possible interactions between audiences and influencers, for instance, in the form of user comments. It may further be relevant for researchers to distinguish between self-generated content and re-posts of other accounts. Capturing the content of interest lastly includes technical considerations regarding data retrieval and data storage, as handling and processing text, image, and video data can be a challenging endeavor.

### Advertisement

With advertisement being the primary source of revenue for most social media platforms and also for influencers, its role in the content produced by political influencers deserves special attention. Traditional influencers, as well as political influencers, can seamlessly integrate a mixture of advertisements and topical content into their social media feeds [3,5]. Such advertisements can come in many forms, such as brand hashtags, unpaid or paid product features, and official partnerships, but many (paid) product recommendations are not officially labeled as such. For political influencers, in particular, advertisements can conflict with authenticity and credibility. When political influencers promote sustainable products or speak out in favor of supporting political parties while receiving monetary compensation at the same time, followers may question whether the content is made up of authentic opinions or merely paid propaganda.

In summary, we highlighted a couple of key factors to be considered for the conceptualization and operationalization of political influencers and the collection of their generated content. As indicated in Fig 1, this is an iterative process in practice, requiring multiple adjustments before finalizing a pipeline for data collection. In what follows, we proceed with a case study demonstrating a quantitative approach to studying political influencers and gaining new insights into their relevance for elections.

## 4. Case study: 2021 federal elections in Germany

Regarding prior work on the federal elections in Germany, 2021 (Bundestagswahl), scholars have used qualitative coding to examine whether influencers’ content includes political knowledge, for instance, about the electoral system in Germany. They found that many influencers offered political education, particularly tailored toward younger target audiences [46]. In comparison, we employ a quantitative empirical approach, seeking to accomplish two goals: first, we demonstrate how to apply the principles mentioned above to study at scale how political influencers interact with major political events, in this case, an election. Second, we provide empirical evidence for the potential impact of political influencers on citizen voting decisions and, therefore, election outcomes.

Regarding Germany, it already became apparent during the European elections in 2019 that German influencers were using their digital reach to tap into the new market of disseminating political content. In May 2019, the influencer “Rezo” published a video on YouTube entitled “The destruction of the CDU”, which was described in international media as the “emergence as the voice of a generation” (Schuetze 2019). The video achieved over 18 million views, and in the video description, the influencer linked to his merchandise shop [47]. In this way, the influencer uses his reach to spread political content while at the same time marketing his products. As outlined above, the development of influencers creating political content comes with both risks and opportunities [3,14].

On the one hand, this can, among other things, potentially increase voter turnout, particularly among younger voters [24]. On the other hand, with advertising as a potential source of income for (political) influencers [5,48–50], monetizing political content makes it difficult to distinguish between authentic opinions and paid propaganda. To evaluate the potential of political influencers, we conduct a case study focusing on the German federal elections in 2021 (Bundestagswahl). In this study, we a) characterize the content produced by political influencers in light of the federal elections and b) provide first insights into their potential for affecting election outcomes.

### Content produced by political influencers

One of the main questions of interest for our case study is whether the dissemination of political posts makes up for the majority of their produced content or is a rather niche phenomenon. While political topics, such as sustainability and environmental protection, can be discussed in isolation, they often reference political actors or events. Especially when it comes to elections, another key question is whether political influencers support or disapprove political entities in the content they produce. At last, we also aim to quantify how much advertisement can be observed in the posts of political influencers, leading to research questions RQ1-RQ3:

**RQ1:** How much political content is produced by political influencers?
**RQ2:** To what extent do political influencers support or disapprove political actors or events?
**RQ3:** How much advertisement is included in the posts of political influencers?

Following the scheme outlined in Fig 1, we first decided on the platform. We focused on Instagram, as it was arguably the most prevalent platform for content creation of political influencers in Germany during this period [46]. We furthermore use a combination of keyword searches (including terms and hashtags with political connotations), network properties (e.g., searching through followees and followers of relevant accounts), and in-depth, qualitative inspections of profiles to identify influencer accounts. Second, for selecting influencer profiles for our sample, we considered the follower count and set our minimum threshold for inclusion at least 10,000 followers. This represents so-called micro influencers [51] but also includes other influencer accounts with higher follower counts. In a third step, we further preselected through a look at the account type. Indications of the account type are, for example, the self-description of the profiles (bio) and categories defined by Instagram, which can, however, also be set up by the profile owners. Fourth, as we are mainly interested in “original” influencers and not just influential accounts, we do not consider politicians or journalists. Rather, we focus on ordinary internet users sharing self-produced details of their lives & subsequently becoming influential by accumulating a large follower base [25], who produce at least some content related to politics. In the final step, we examine whether profiles have uploaded content with paid partnerships or advertising. Our final sample includes 98 accounts of political influencers who match our criteria. After identifying our main sample of interest, we used the Crowdtangle API [52] combined with additional data scraping procedures to capture the content produced by all accounts. We first use Crowdtangle API daily to get information about all posts produced by influencers. In the second step, we scrape videos, images, and photos of all posts. The data was collected between June 2021 and September 2021. We extracted a total of 3.164 posts, which were liked by around 30 million people and received around 335,000 comments at the end of our data collection period. Our data not only allows us to provide a one-time categorization of influencer content but also to identify time trends and evaluate influencers to adjust their posts in light of the federal elections.

However, a significant challenge is that our concepts of interest are notoriously difficult to measure. This is due to several reasons: First, the Instagram content is multimodal, consisting of text, photos, and videos. Despite several advances in machine learning and artificial intelligence for multimodal data [53], it is still difficult to identify multiple concepts across data types. Second, our concepts of interest cannot be captured via simple procedures. For one, there is no straightforward mapping from a definition to the operationalization of what can be considered political content - particularly, in a broad sense, covering a vast range of topics.

Moreover, while Instagram has integrated platform features for declaring paid sponsorships, these features are used only very infrequently. In combination, these challenges restrict methods for fully automated concept detection. For this reason, we employ crowdsourcing for human-assisted coding, a concept that has been applied successfully for coding political data in the past [54]. We use the platform Prolific to recruit a sample of German-speaking raters with a balanced gender ratio. Regarding the quality of data obtained via crowdsourcing, recent work has highlighted that a substantial share of crowd workers use Large Language Models for task completion [55]. While we can never fully rule out biases in our data, this concern is very unlikely to affect our task, as models were not nearly as proficient nor as easy to use as recent models at the time of data collection in 2021. This applies particularly to working with image data, a crucial component of our task. In addition, Prolific has been found to provide data with higher quality than other providers and is explicitly designed for scientific research [56]. For our task, we recruited a total of 357 participants and also paid above-average rates compared to other tasks on the platform with similar time investment.

Using the R-Shiny framework [57], we created a web application for the coding procedure. For each coder, a random subset of posts was shown. In addition to text, we showed up to four images for multi-image posts and the video thumbnail for video posts. For each post, coders were asked to indicate whether it includes political content (RQ1), support or disapproval of political actors (RQ2), and/or advertisement (RQ5). Our related coding instructions, as well as several example posts covering each of the categories, are available in the supplementary materials **S1** and **S2** in S1 Text. For each post, we accumulate at least three different coders, resulting in about 10,700 total ratings. In the last step, we identify the majority mode of codings for each category among coders and discard posts without modal agreement. We calculated these shares of posts without a modal agreement for each category: ~ 5% for support or disapproval of political entities, ~ 2% for advertisement, and ~ 1% for political content. In other words: we have a majority vote for 95% of posts related to support or disapproval of political actors, 98% related to advertisement, and 99% related to political content. These numbers suggest reasonable agreement between coders. Through qualitative examination, we found that the discarded posts were predominantly ambivalent with little meaning and thus not relevant to our case study. This procedure resulted in a final sample of 2.915 posts, 92% of our initial sample. At the time of data collection, these posts generated a cumulate reach of about 312 thousand comments and 29 million likes on Instagram.

### Potential for affecting election outcomes

While the content produced by political influencers in the run-up to the election provides valuable insights into political influencers, analyzing it in isolation is only of limited use for evaluating the relevance of election outcomes. First, it is important to determine which kind of voters are likely to be in contact with influencers’ content in the first place. Second, consuming influencer content does not necessarily affect personal voting decisions. In addition, it is important to understand whether the content of influencers provided useful information for the decision-making of voters. This leads us to our research questions RQ4-RQ5:

**RQ4: Which voters are following the content of influencers?**
**RQ5: To what extent do voters consider influencers’ content relevant to their election-related decision-making?**

To answer these questions, we collaborated with the Online Access Panel provider Respondi to run a follow-up survey for the German federal elections. We restricted the participant pool to people eligible to vote and introduced sampling quotas by age, sex, education, and federal state according to the German census. In our supplementary material **S4 in** S1 Text, we compare the socio-demographic composition of our sample to the census. As expected, our sample composition regarding sex, age, education, and federal state, is well-aligned with the target population of German citizens. Our survey was conducted roughly three weeks after the election, with a field time from October 19 to October 25 and a total of 1.107 participants. Informed consent was acquired via the access panel provider. For our questionnaire, we mostly relied upon items from other large surveys, in particular, the German Longitudinal Election Study (GLES) [58], and added influencer-related questions. An overview of data quality checks is included in our supplementary material **S5** in S1 Text. We compared voting shares from our survey with those from the GLES Post-Election Study and the actual results of the federal election. We find a high similarity between voting shares from our survey and those of both GLES and the election results. At last, we compared self-collected participant demographics to those provided by Respondi and removed a few speeders, resulting in a final sample of 1.059 survey participants. As speeders, we define all participants who either did not complete the questionnaire in full or took less than 120 seconds to complete it. Note that including or excluding speeders has no substantial effect on our results [59].

## 5. Case study results

**Fig 2:**
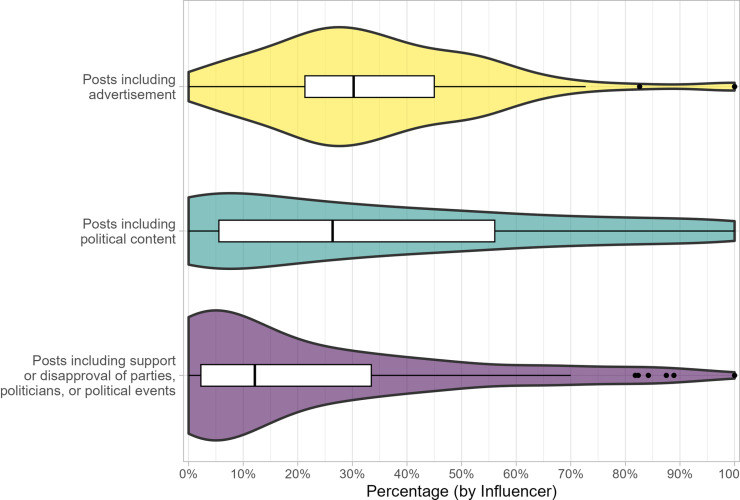
Content produced by political influencers on Instagram. Percentages denote the share of posts for each influencer in our sample coded as belonging to either category. Example interpretation: The median account includes advertisements in about 31% of all posts.

To quantify the kind of content political influencers post on Instagram (RQ1 - RQ3), we rely on relative measures, as the number of posts can vary substantially across accounts. For each influencer, we compute the share of posts, including advertisements, political content, and the support or disapproval of political entities. Fig 2 depicts the corresponding percentages using a violin plot. As can be seen, a substantial number of posts include political content, although the share for advertising is even higher. The median influencer includes advertisement in about 31% of all posts and political content in about 27%. In comparison, support or disapproval of parties, politicians, or political events is less prevalent at about 12% but still noticeable. In our supplementary material **S3** in S1 Text, we include additional analysis showing that the post type does affect the number of received likes and comments, where support or disapproval of political entities increase, and advertisements rather decrease received attention on Instagram.

**Fig 3:**
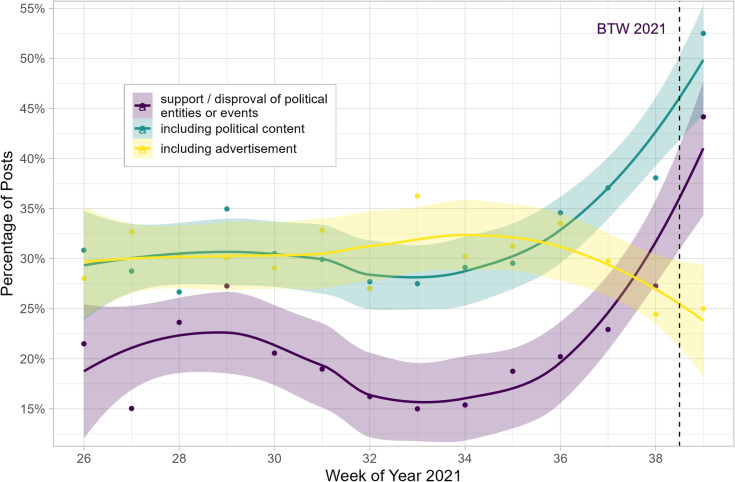
Weekly percentages for content types of posts by political influencers. Points denote average weekly values, and bands denote loess-smoothed uncertainty estimates. Example interpretation: In week 26, about 29% of posts included political content and advertisements compared to about 18%, including support or disapproval of political entities and events.

Next, we examine whether political influencers adjust their content dynamically over time and opt for different content strategies as the federal election approaches. Fig 3 includes weekly averages of percentages for our content categories, including uncertainty bands estimated with a LOESS (locally estimated scatterplot smoothing) algorithm. It becomes apparent that influencers adjust the content they produce during our observation period in light of the federal election. In the earlier weeks, advertisements are at or above the share of political content, whereas they are used substantially less during the weeks before the election. In comparison, the share of political content and support or disapproval of political entities increased during the weeks before the election.

**Fig 4:**
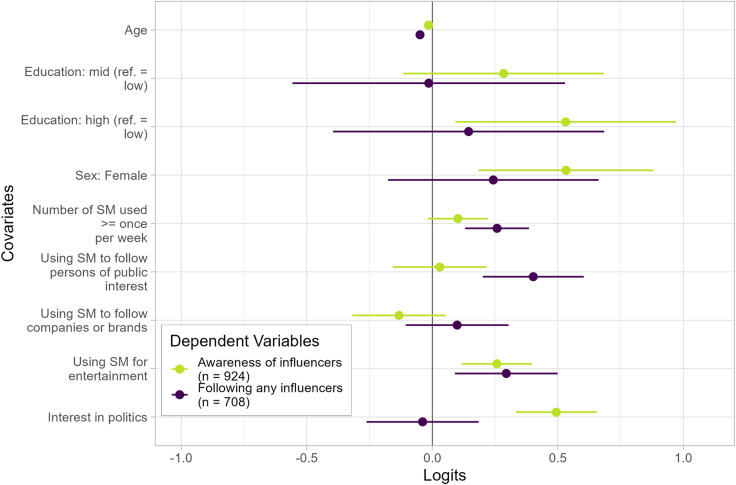
Socio-demographics and item responses of survey participants and their association with being aware of or following influencers. Points depicted in the forest plot denote logit coefficients from two logistic regression models (one for each dependent variable). Bars denote 95% confidence intervals.

We now turn to the results of our survey of German citizens to evaluate the potential for affecting election outcomes. First, we use two logistic regressions to analyze the associations between several respondents’ characteristics and whether they are aware of or follow the content of influencers on social media (RQ4). Corresponding Forest plots for both models, including 95% confidence intervals, are depicted in Fig 4. It becomes apparent that participants of lower ages are likelier to be aware of or follow influencers’ content, as indicated by the negative logits. In addition, higher educated (in comparison to lower educated) and female (in comparison to male) participants are more likely to be aware of influencers. The higher the interest in politics, the likelier it is for participants to be aware of influencers. However, neither sex, education, or interest in politics as associated with the likelihood of following influencers. Furthermore, we find mixed results regarding the use of social media but a positive association between the use of social media for entertainment and both dependent variables.

**Fig 5:**
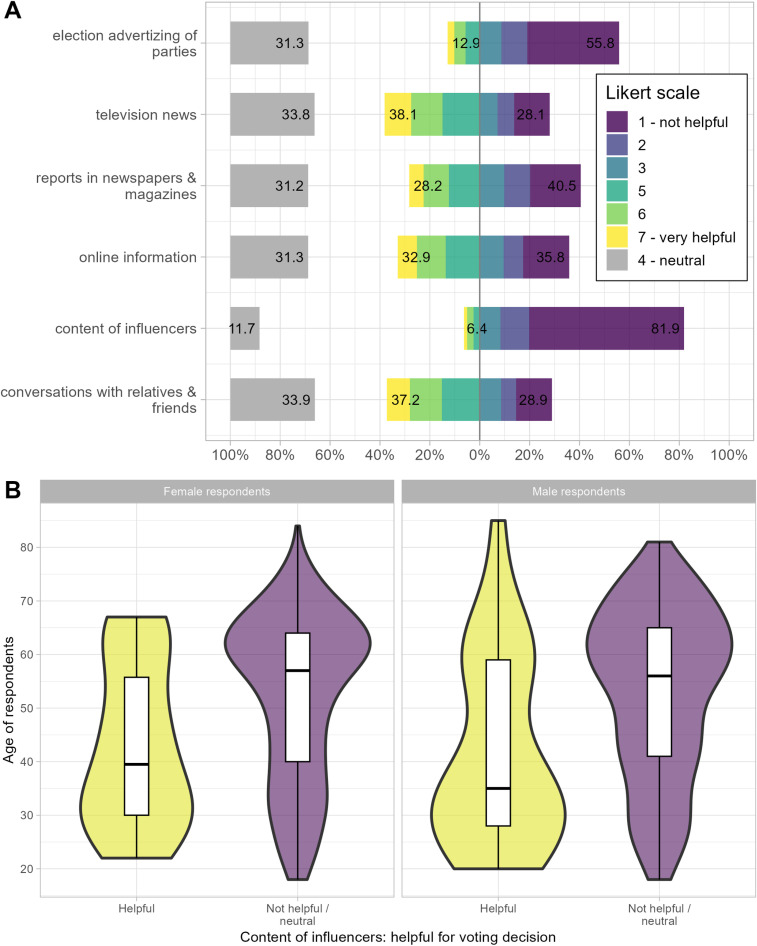
Helpfulness of influencer content for voting decisions of survey respondents. Panel **A.** Various items for how helpful factors were in determining their voting decisions for the federal election. The Likert scales range from 1 - not helpful to 7 - very helpful, with 4 representing the neutral category. Numbers indicate the cumulative percentages for neutral, helpful, and unhelpful indicators. Example interpretation: 6.4 percent of respondents indicated that influencers’ content helped determine their voting decision, whereas 38.1 percent found television news helpful. Panel **B.** Violin plots for respondent age by sex and respondents’ dichotomized answer to how useful influencer content is for their voting decisions. Example interpretation: the median age of male respondents who do not find influencer content helpful for their voting decisions is 56, while the median age of male respondents who find this content helpful is 35.

To address our last research question about whether influencers can affect voting decisions (RQ5), Panel A of Fig 5 visualizes the shares of response categories for various survey items. Across all items, participants were asked to rate how helpful these factors were in determining their voting decisions for the federal election on a Likert scale ranging from 1 - not helpful at all one end to 7 - very helpful, and 4 represents the “neutral” category. In line with results from other German election studies [58], conversations with friends and relatives, television news, and online information are among the most helpful sources for determining voting decisions. About 37% of participants considered discussions with friends and relatives to be helpful, in comparison to a low share of about 6% who considered the content of influencers as helpful. In relative terms, one might ultimately conclude that influencers, if anything, play a marginal role in affecting voting decisions. However, in absolute terms, 6% of the electorate in Germany would nevertheless translate to numerous people for whom influencers’ content matters for their voting decisions.

Lastly, the question arises of what kind of participants consider influencers when making their voting decisions. Panel B of Fig 5 includes violin plots differentiating between age, sex, and the indicator for helpful influencer content (values 5–7 on the Likert scale). It becomes apparent that age plays an important role in particular. For instance, the median age of male respondents who do not find influencer content helpful for their voting decisions is 56, while the median age of male respondents who find this content helpful is 35. In comparison, if anything, sex is very weakly associated with finding influencer content helpful or not helpful for voting decisions. In our supplementary material **S6** in S1 Text, we further examine whether the small share of people who indicated influencers’ content to be helpful voted differently than the other voters, and find no substantial differences between these groups.

## 6. Conclusions

With the overall spread of influencers on social media, political influencers have also increasingly established themselves and are shaping political communication online. In addition to influencers who traditionally deal with lifestyle topics, they are involved in political advocacy and discourse [3]. We addressed this development by highlighting key factors for the conceptualization and operationalization of political influencers and provided new insights into their impact on elections in our case study. One of the key findings from our study is that political influencers engage in a dynamic blend of content, often integrating political messaging with product promotions. However, as the election approaches, their focus shifts toward political content and support or disapproval of political actors, aligning with findings from previous studies that indicate political topics are most often shared in the form of commentaries or reaction videos [6]. On that end, it is important to note that our sample was constructed to focus on influencers who regularly post political content. Additionally, our data collection took place during the time of the election campaign, in which an increased proportion of political posts was to be expected. Both of these factors are likely to be associated with the high percents of political content in influencers’ posts we find in our analysis. In comparison, other studies report lower percentages [12].

Our results about participants of lower ages being more likely to follow influencers is in line with expectations, as prior research has found that large shares of younger cohorts are following influencers on social media [60]. Findings from our survey further show that using social media for entertainment predicts following and being aware of influencers. On the one hand, this suggests that influencers may reach audiences primarily seeking entertainment rather than political content and therefore might be able to increase political participation among hard-to-reach groups. On the other hand, some studies show that such incidental exposure has only limited effects in political knowledge and participation in general [61, 62]. This raises questions about the extent to which political influencers fulfil democratic hopes of engaging disengaged audiences in meaningful political discourse, which is an important avenue for future research.

Results from our survey also suggest that the impact on individual voting decisions of political influencers may appear modest at first, especially when compared to more traditional sources of influence, such as conversations with family and friends. Nevertheless, with 6% of survey participants - particularly younger citizens - reporting that influencer content was helpful in their decision-making, this small fraction represents a substantial share of the electorate in Germany. In competitive elections, such as the 2021 Bundestagswahl, even minor shifts in voter behavior influenced by political influencers could alter the final result. However, our survey data does not allow us to infer the direct impact of influencer content on electoral outcomes, as we cannot identify causal effects of factors being helpful for voting decisions on the actual voting behavior of respondents.

Regarding other limitations of this work, our data was constrained to Instagram as the primary social media platform. Future studies could benefit from expanding the scope to include multiple social media platforms, thereby capturing a more diverse range of political influencers and their audiences. Second, while our crowdsourced content analysis provided valuable insights into the political and commercial content shared by influencers, identifying political messages in multimodal content (such as images, videos, and text) remains a significant challenge. Despite advances in machine learning, fully automated content detection remains limited, and our reliance on human coders introduced potential subjective biases. In our replication material, we provide our codings for future research, which can be used as training material for machine-learning approaches to better capture and quantify multimodal political content.

While our combination of analyzing social media content and post-election survey data provides new insights, it was not possible to link both data sources. Combining digital trace data with surveys so that participants can be questioned about their observed online behavior would allow the investigation of many more important questions and is another promising avenue for future research. Another important direction for future studies lies in the cross-national comparison of political influencers’ effects on elections. Lastly, while our case study focused on the 2021 German federal elections, political influencer dynamics may vary significantly across different political systems, cultures, and levels of social media saturation. Exploring these differences could help to quantify the broader impact of influencers on global electoral outcomes and political discourse.

Regarding our survey, we used the broader and at the time of data collection more widely recognized term “influencers” to ensure clarity and accurate responses, as opposed to “political influencers”. At the same time, our survey strongly induced a political framing throughout the process for our participants via introductory texts, item wordings, and other means. However, we still cannot be certain about what respondents had in mind when answering survey questions. Although we consider it unlikely, it is possible that they did not mainly consider influencers posting political content when responding to our related items. Future research could shed light on how to improve newly developed survey items for influencers using methods such as cognitive interviewing [63], which was beyond the scope of this study.

In conclusion, while political influencers are still a relatively new phenomenon in the political landscape, their growing importance in shaping political discourses should not be ignored [3,38]. As Harff and Schmuck [12] highlight, political content shared by influencers can garner significant engagement, pointing to their potential to amplify political messages. At the same time, the interplay between their commercial and political activities raises questions about authenticity and trustworthiness [4]. As platforms increasingly limit the visibility of political content [64], it remains to be seen how political influencers adapt to these challenges while maintaining their status as trusted opinion leaders.

## Supporting information

S1 Text S0 File. Reproduction Material. S1 File. Crowdsourcing for coding the content produced by political influencers. S2 File. Example Post to be coded by crowdsourcer. S3 File. Associations between codings and popularity metrics. S4 File. Post-Election Survey: Quota sample. S5 File. Post-Election Survey: Quality Checks. S6 File. Post-Election Survey: Vote Share Comparison by Influencer Helpfulness. (PDF)
