# Supplementary material for: From hashtags to ballots: Conceptualizing political influencers and evaluating their impact on election outcomes
Source: PLoS One. 2025 May 7;20(5):e0321592. doi: 10.1371/journal.pone.0321592 (PMC12143925; doi:10.1371/journal.pone.0321592)
Supplement: S1 Text — (PDF) [file pone.0321592.s001.docx]

**Supplementary Material**

“From Hashtags to Ballots: Conceptualizing Political Influencers and Evaluating Their Impact on Election Outcomes”

**S0 - Reproduction Material**

We created a data repository at Harvard Dataverse which includes all necessary files for reproducing our results for the main paper and this supplementary document: <https://doi.org/10.7910/DVN/QMXOPK>

*Software*

For our analysis, we used several R packages and related software: R version 4.4.1 (R Core Team 2024) and the following R packages: cowplot v. 1.1.3 (Wilke 2024), hrbrthemes v. 0.8.7 (Rudis 2024), lme4 v. 1.1.35.5 (Bates et al. 2015), sjlabelled v. 1.2.0 (Lüdecke 2022), sjmisc v. 2.8.10 (Lüdecke 2018), sjPlot v. 2.8.16 (Lüdecke 2024), tidyverse v. 2.0.0 (Wickham et al. 2019).

Bates, Douglas, Martin Mächler, Ben Bolker, and Steve Walker. 2015. “Fitting Linear Mixed-Effects Models Using lme4.” Journal of Statistical Software 67 (1): 1–48. <https://doi.org/10.18637/jss.v067.i01>.

Lüdecke, Daniel. 2018. “sjmisc: Data and Variable Transformation Functions.” Journal of Open Source Software 3 (26): 754. <https://doi.org/10.21105/joss.00754>.

Lüdecke, Daniel. 2022. sjlabelled: Labelled Data Utility Functions (Version 1.2.0). <https://doi.org/10.5281/zenodo.1249215>.

Lüdecke, Daniel. 2024. sjPlot: Data Visualization for Statistics in Social Science. [https://CRAN.R-project.org/package=sjPlot](https://cran.r-project.org/package=sjPlot).

R Core Team. 2024. R: A Language and Environment for Statistical Computing. Vienna, Austria: R Foundation for Statistical Computing. [https://www.R-project.org/](https://www.r-project.org/).

Rudis, Bob. 2024. hrbrthemes: Additional Themes, Theme Components and Utilities for “ggplot2”. [https://CRAN.R-project.org/package=hrbrthemes](https://cran.r-project.org/package=hrbrthemes).

Wickham, Hadley, Mara Averick, Jennifer Bryan, Winston Chang, Lucy D’Agostino McGowan, Romain François, Garrett Grolemund, et al. 2019. “Welcome to the tidyverse.” Journal of Open Source Software 4 (43): 1686. <https://doi.org/10.21105/joss.01686>.

Wilke, Claus O. 2024. cowplot: Streamlined Plot Theme and Plot Annotations for “ggplot2”. [https://CRAN.R-project.org/package=cowplot](https://cran.r-project.org/package=cowplot).

**S1 - Crowdsourcing for coding the content produced by political influencers**

As outlined in the main paper, we used crowdsourcing via the Prolific platform to code the content produced by political influencers on Instagram. In what follows, we provide additional details on the crowdsourcing procedure.

*Information for participants before participating in the crowdsourcing task (in German language)*


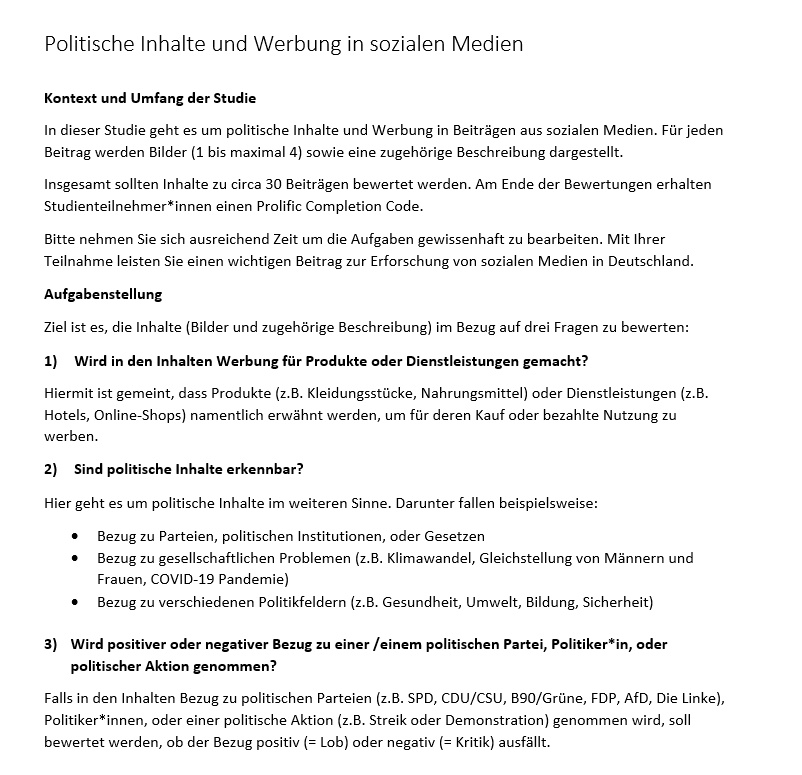


***Figure S1.***  *Coding instructions for crowd workers in the German language*

**S2 - Example Post to be Coded by Crowdsourcers**

The following image shows an example post for crowdsourcing to be coded during the task. They are provided with the first (up to a maximum of four) images of a post and the corresponding textual description. Afterward, they are asked to indicate whether the posts include a) advertising, b) political content, and or c) support or disapproval related to political actors or political events.


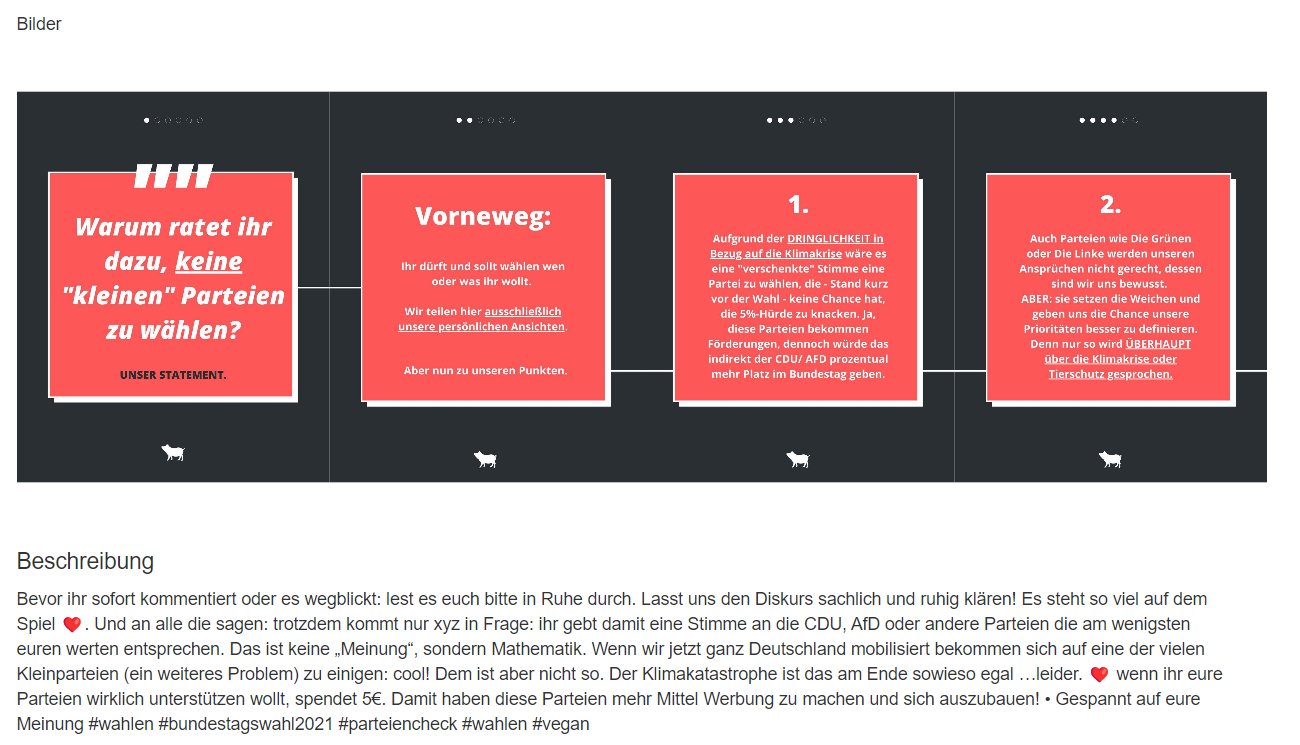

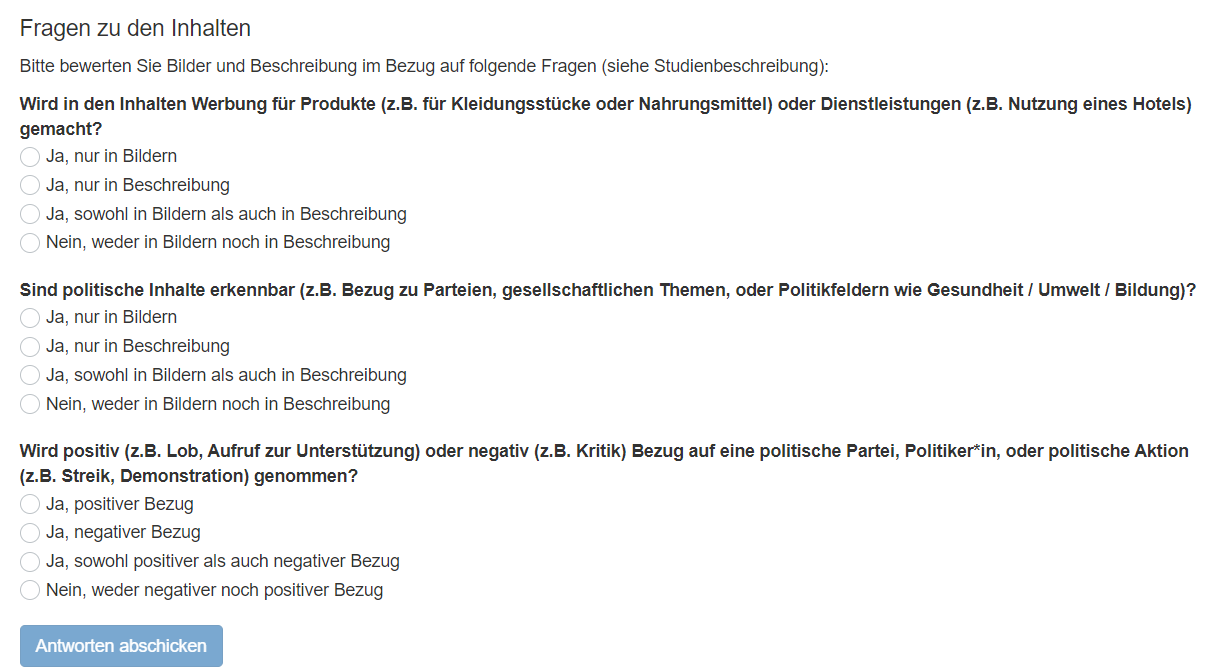


***Figure S2.***  *Example Post to be Coded by Crowdsourcers (in the German language)*

**S3 - Associations between codings and popularity metrics**

We used multilevel negative-binomial regression models, where posts are nested into influencer accounts, to analyze whether the crowdsourced coding of Instagram posts—alongside tagging other users in a post—is associated with an increase or decrease in a post’s popularity. We measure popularity with two dependent variables: the likes and comments a post receives during data collection. As seen in the forest plot below, support and disapproval of political entities are both associated with the increasing popularity of posts. The signal is less clear when just including political content, which stipulates more user comments but is not associated with a higher number of received likes. In comparison, including advertisement and product promotion reduced the popularity of posts, whereas tagging users does not have a substantial effect.


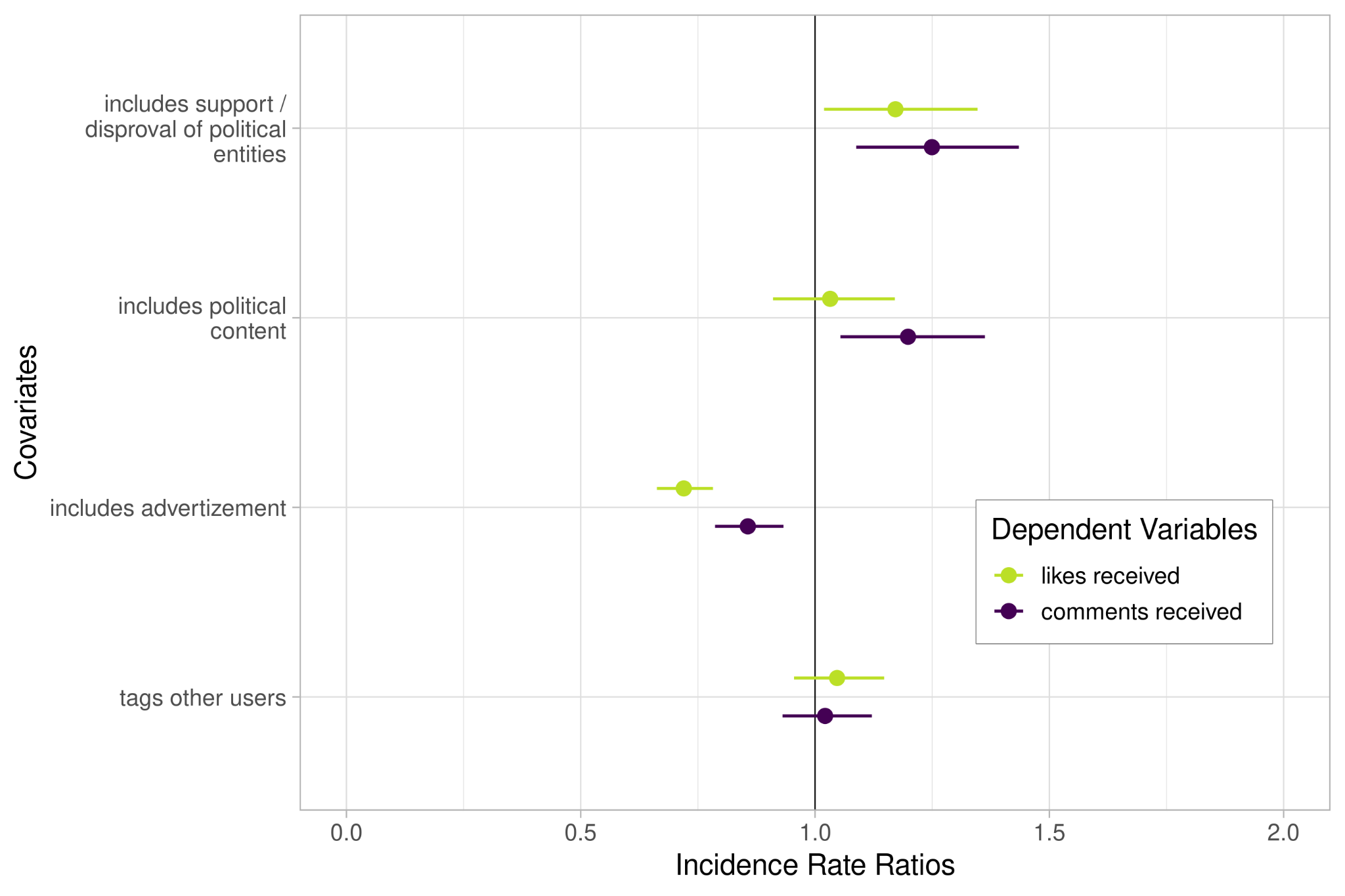


***Figure S3.*** *Forest plot showing the effect of content type on the popularity of Instagram posts for both likes and comments. Multi-level negative binomial models with varying slopes for different influencers. Depicted are point estimates and 95% confidence intervals.*

**S4 - Post-Election Survey: Quota sample**

For our post-selection survey, we used quota data from the German census to create a sample of persons who closely matched the target population (German citizens eligible to vote). The following visualization depicts the distribution of survey participants by age group and sex:


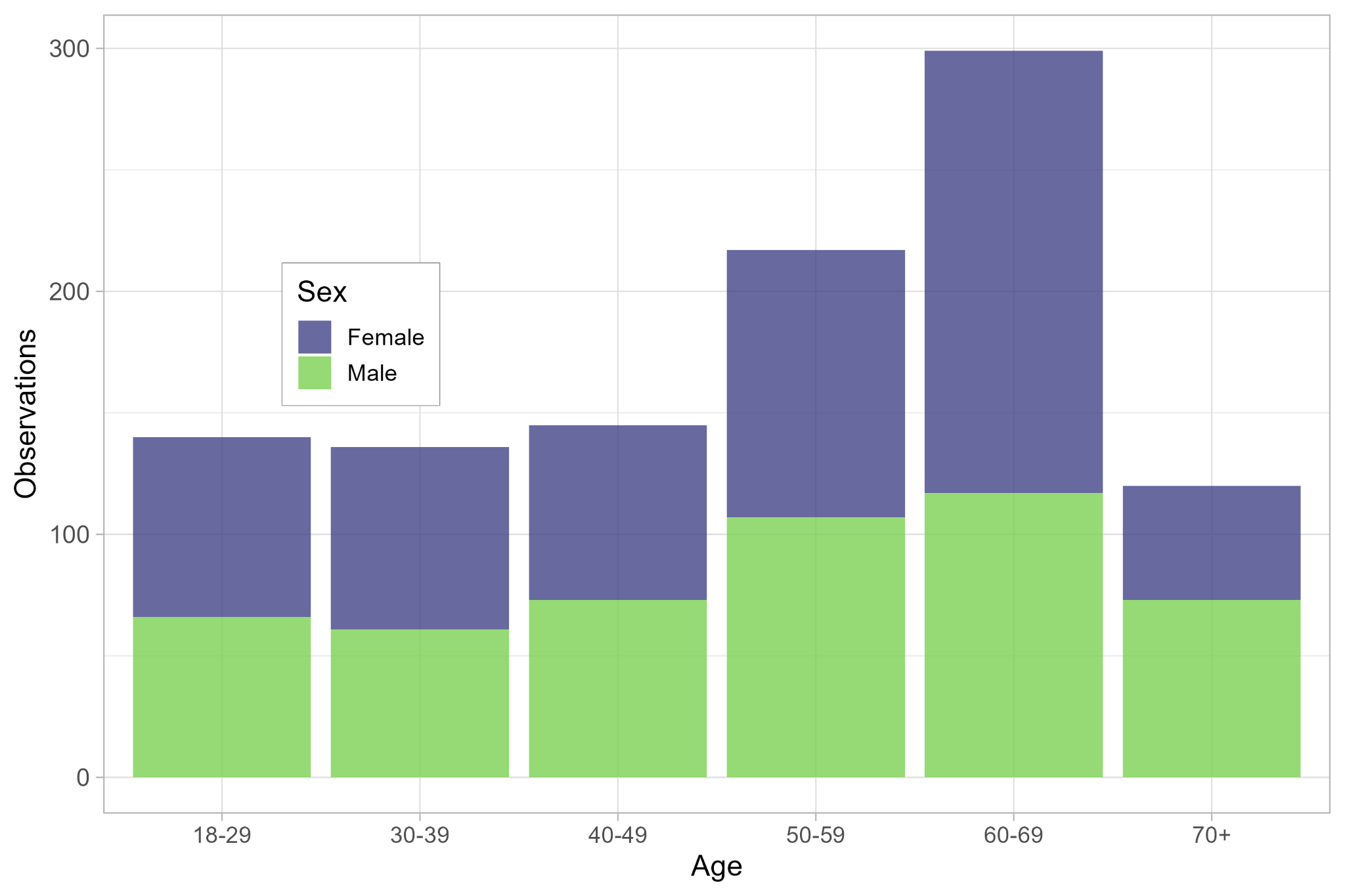


**Figure S4.** Distribution of age and sex for respondents of our survey.

For our survey, we introduced quotas along three criteria: a combined indicator for age and xex, place of residence (federal country), and participants' education (categorized into lower, mid, and high education). The following tables provide an overview of expected shares according to the German census compared to the actual share in our survey sample. As can be seen, the distribution in our sample is (by design) well-aligned with the German census data.

| **Sex & Age** | **Expected Share** | **Survey Share** |
| --- | --- | --- |
| m 18-29 | 7.5 | 6.2 |
| m 30-39 | 7.3 | 5.8 |
| m 40-49 | 6.8 | 6.9 |
| m 50-59 | 9.9 | 10.1 |
| m 60+ | 16.9 | 17.9 |
| f 18-29 | 7.2 | 7 |
| f 30-39 | 7.1 | 7.1 |
| f 40-49 | 6.8 | 6.8 |
| f 50-59 | 9.9 | 10.4 |
| f 60+ | 20.7 | 21.6 |

| **Education (Categorical)** | **Expected Share** | **Survey Share** |
| --- | --- | --- |
| Low education | 31.1 | 31.6 |
| Mid education | 31.4 | 31.9 |
| High education | 37.5 | 36.4 |

| **Federal State** | **Expected Share** | **Survey Share** |
| --- | --- | --- |
| Berlin | 4.1 | 4.2 |
| Brandenburg | 3.3 | 3.5 |
| Mecklenburg-Vorpommern | 2.1 | 2.2 |
| Sachsen | 5.2 | 5.5 |
| Sachsen-Anhalt | 2.9 | 3 |
| Thüringen | 2.8 | 2.8 |
| Baden-Württemberg | 12.7 | 12.1 |
| Bayern | 15.6 | 15.6 |
| Bremen | 0.8 | 0.9 |
| Hamburg | 2.1 | 1.8 |
| Hessen | 7.2 | 6.7 |
| Niedersachsen | 9.9 | 9.9 |
| Nordrhein-Westfalen | 21.3 | 21.6 |
| Rheinland-Pfalz | 5.0 | 5.1 |
| Saarland | 1.2 | 1.3 |
| Schleswig-Holstein | 3.7 | 3.8 |

***Table ST1.*** *Expected and actual shares for socio-demographics of survey respondents. Expected shares are derived from German Census data.*

**S5 - Post-Election Survey: Quality Checks**

To ensure the quality of our post-election survey was high, we conducted several quality checks. First, we compared participants' demographics as provided by the company (respondi) managing the participant pool with those captured in our survey. We found no substantial difference. Second, we removed participants from our sample who were unreasonably fast when finishing the survey. In our case, this corresponded to a completion time of less than two minutes. This procedure removed about 4% of participants and resulted in a final n = 1059. Third, we compared the voting shares from the 2021 German federal elections with the voting shares of participants of a major Post-Election survey (GLES) and our own survey. As can be seen in the following figure, voting shares for most parties are quite similar for most parties.


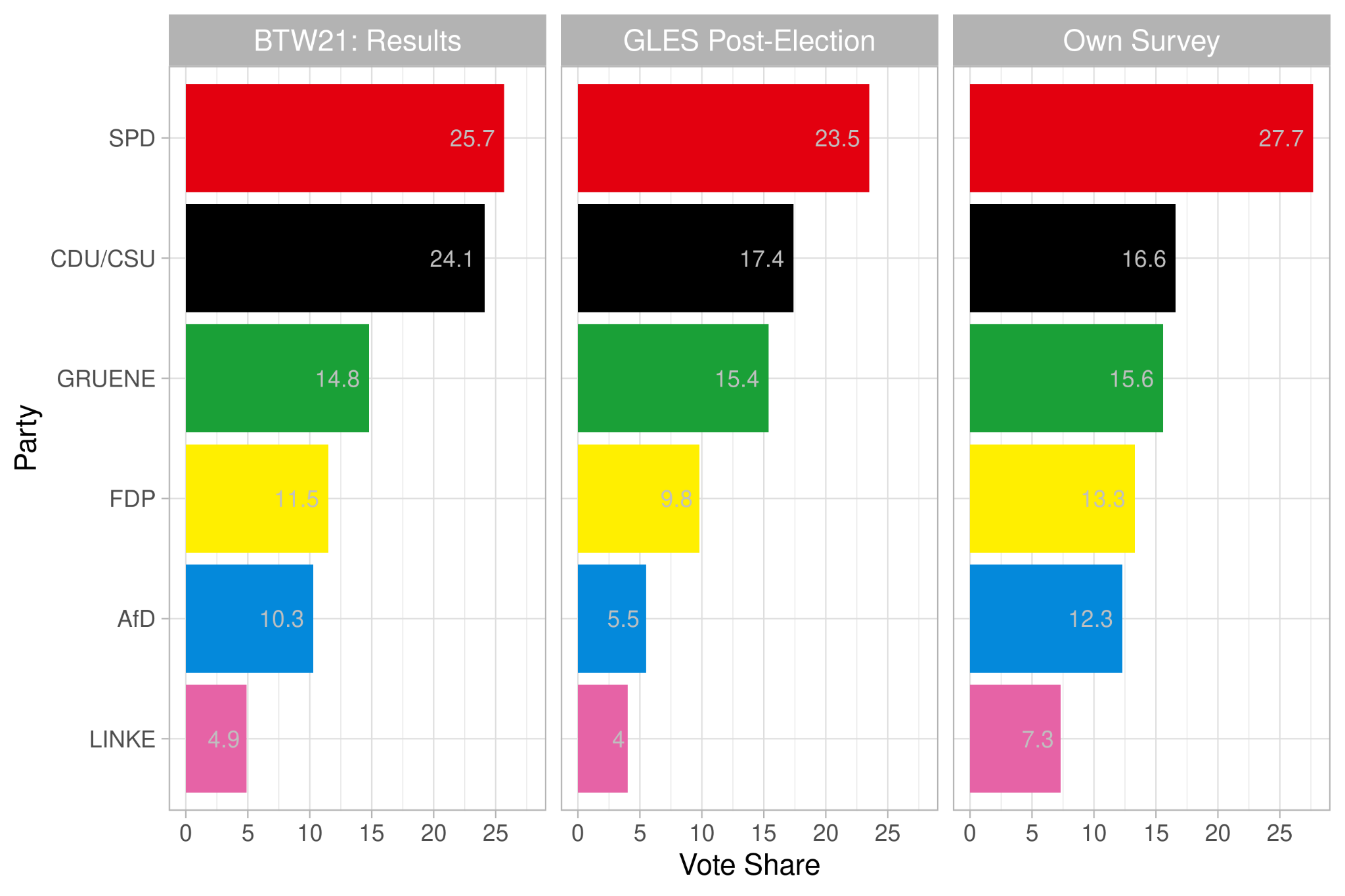


**Figure S5. Vote shares from different sources.**

**S6 - Post-Election Survey: Vote Share Comparison by Influencer Helpfulness**

In our main paper, we show Likert items for factors that were to varying degrees helpful for participants in making up their voting decisions. In the figure below, we compare voting shares between those who found influencers helpful or not helpful. Minor differences become apparent, for instance, a higher voting share for the German Green party (“DIE GRÜNEN”) and a lower share for the right-wing party Alternative für Deutschland (“AFD”).


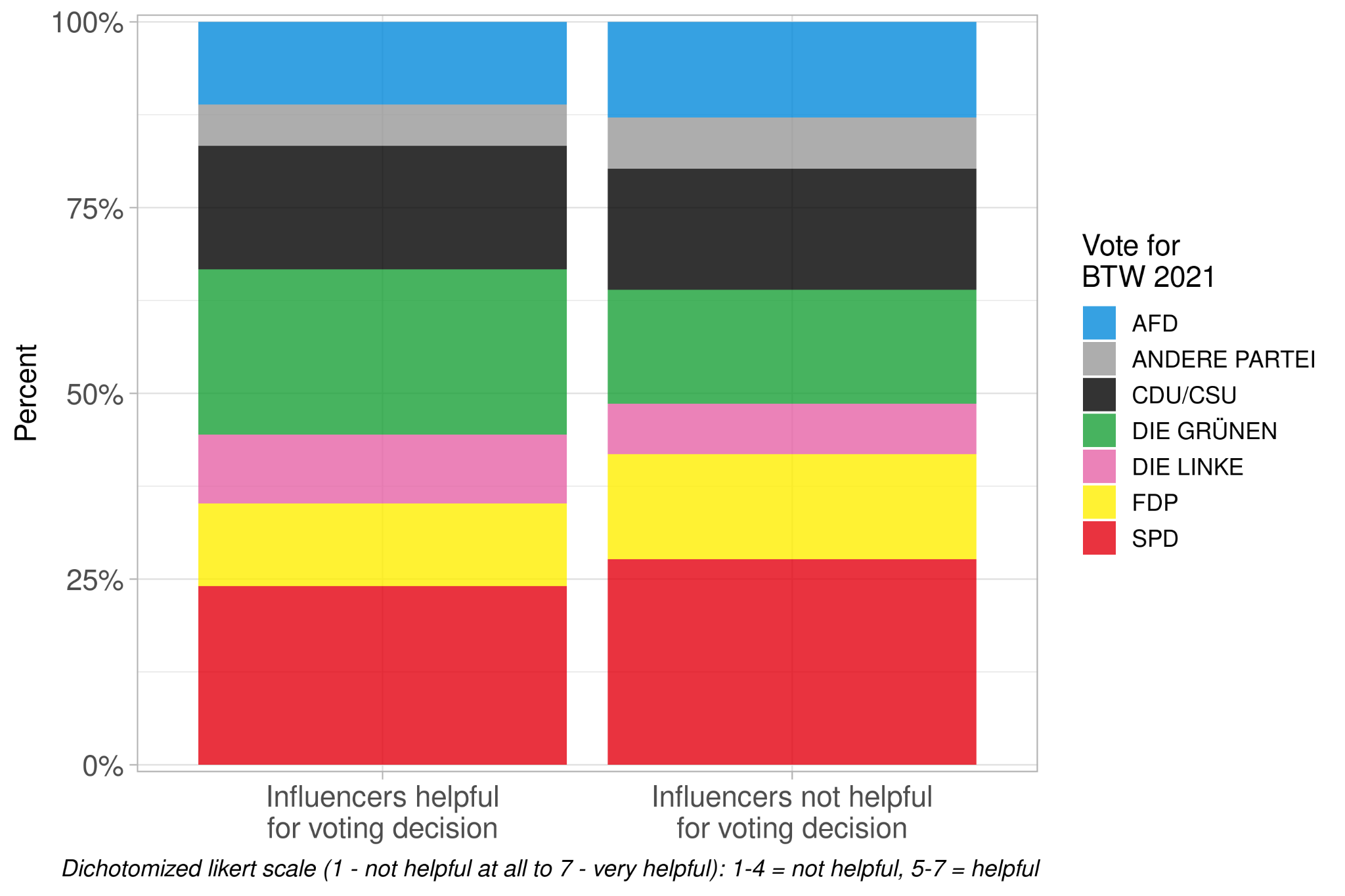


**Figure S6. Comparison of voting shares by the helpfulness of influencer content for voting decisions.**
